# Barriers to Adoption of Electronic Low Vision Aids Among Eye Care Professionals in Jordan: Descriptive Cross-Sectional Study

**DOI:** 10.2196/87685

**Published:** 2026-03-02

**Authors:** Areej Okasheh-Otoom

**Affiliations:** 1 Faculty of Applied Medical Sciences, Jordan University of Science and Technology (JUST), PO Box 3030, Irbid, 22110, Jordan, 962 27201000

**Keywords:** low vision, electronic low vision aids, assistive technology, digital rehabilitation, adoption barriers, Jordan, optometry, ophthalmology

## Abstract

**Background:**

Digital, smart, and electronic low vision aids (LVAs) have expanded options for visual rehabilitation and functional independence among people with visual impairment. However, adoption of these technologies remains limited, particularly in low- and middle-income countries such as Jordan, where access, affordability, and training resources may be constrained.

**Objective:**

To examine barriers to the adoption of electronic LVAs and identify factors associated with their use among eye care professionals in Jordan.

**Methods:**

A descriptive cross-sectional survey was conducted among 270 eye care professionals working in hospitals, rehabilitation centers, and private clinics across Jordan. The questionnaire assessed awareness, training exposure, institutional support, and perceived barriers related to electronic LVAs. Descriptive statistics and inferential analyses were used to examine adoption patterns and predictors, with statistical significance set at *P*<.05.

**Results:**

Of the 270 participants, 156 (57.8%) were optometrists, 78 (28.9%) were ophthalmologists, and 36 (13.3%) were low vision specialists. The mean age was 36 (SD 8) years, and the mean professional experience was 12 (SD 6) years. Overall, 117 of 270 (42.2%) participants reported current use or recommendation of electronic LVAs. The most frequently reported barriers were high device cost (n=213, 79%), lack of training (n=184, 68.1%), limited institutional support (n=173, 64%), and low patient awareness (n=154, 57%). In multivariable analysis, greater training exposure (odds ratio [OR] 1.82, 95% CI 1.31‐2.53; *P*<.001), stronger institutional support (OR 1.48, 95% CI 1.12‐1.96; *P*=.008), and higher awareness scores (OR 1.35, 95% CI 1.05‐1.72; *P*=.02) were positively associated with aid adoption, whereas high device cost was negatively associated with aid adoption (OR 0.41, 95% CI 0.27‐0.62; *P*<.001).

**Conclusions:**

Adoption of electronic LVAs among eye care professionals in Jordan remains limited. Cost, training exposure, and institutional support are key factors influencing uptake. These findings suggest that strengthening professional training and institutional support may facilitate broader integration of electronic LVAs into low vision rehabilitation services.

## Introduction

Visual impairment affects more than 2.2 billion people worldwide, and at least 1 billion cases could have been prevented or remain unaddressed [1]. Low vision rehabilitation aims to enhance residual vision and functional independence using optical and electronic assistive technologies [2]. Recent advances in portable, digital, and smart low vision aids (LVAs), including magnifiers, wearable displays, and smartphone-based systems, have significantly improved navigation and access to information [2-5]. These devices, collectively termed electronic LVAs, complement traditional optical aids by offering adjustable magnification, contrast enhancement, and speech feedback [6,7].

Despite growing evidence supporting their functional and psychosocial benefits [8-11], adoption of electronic LVAs remains limited globally [12]. Reported barriers include high device cost, inadequate professional training, limited awareness among clinicians and patients, and insufficient institutional and policy support [13-15]. These challenges are particularly pronounced in low- and middle-income countries, where limited service infrastructure, fragmented referral pathways, and absence of sustainable funding mechanisms further restrict access to electronic low vision technologies [2].

In Jordan, visual impairment represents the most prevalent form of disability, affecting approximately 6% to 7% of the population aged 5 years or older, according to national statistics [16]. Although optometric education and eye care infrastructure have expanded in recent years, integration of structured low vision rehabilitation services within the health care system remains inconsistent [7,15,17]. Available local evidence indicates that assistive and digital technologies are used predominantly in educational or professional contexts, with limited incorporation into formal low vision rehabilitation pathways. Regional studies further suggest that fewer than half of eye care professionals routinely prescribe or recommend electronic LVAs, underscoring persistent gaps in training, access, and service delivery [3,18,19]. Understanding context-specific barriers in Jordan is therefore essential for developing targeted professional training and service-level interventions.

Technology adoption frameworks, including the technology acceptance model and the unified theory of acceptance and use of technology, provide a useful lens for examining these challenges [20]. These models emphasize perceived usefulness, ease of use, and facilitating conditions as key determinants of adoption [21]. In low vision rehabilitation, these constructs translate into clinician awareness, perceived patient benefit, affordability, and availability of institutional support. While prior studies have examined assistive technology use in various contexts, limited evidence exists regarding how these adoption factors interact among eye care professionals involved in low vision care in Jordan. The aim of this study was to examine awareness, training exposure, institutional support, perceived barriers, and factors associated with the adoption and recommendation of electronic low vision aids among eye care professionals in Jordan.

## Methods

### Study Design

A descriptive cross-sectional study was conducted among eye care professionals working in public and private hospitals, university eye clinics, and vision rehabilitation centers across Jordan. This study was implemented as an online cross-sectional survey and is reported in accordance with the CHERRIES (Checklist for Reporting Results of Internet E-Surveys) guidelines (Checklist 1). Invitations were distributed electronically through established professional networks, including national professional associations, institutional mailing lists, and closed professional communication groups (eg, email lists and secure messaging platforms) commonly used by ophthalmologists, optometrists, and low-vision specialists in Jordan. These networks included clinicians working in public, private, and academic settings across Jordan. Distribution was not stratified by geographic region or sector; therefore, participation depended on individual access to and engagement with these professional channels. Survey items were presented in a fixed order. Completion of all mandatory closed-ended items was required for submission, whereas open-ended questions were optional. Only fully completed questionnaires were included in the analysis.

Eligible participants included optometrists, ophthalmologists, and low-vision specialists currently practicing in Jordan. Inclusion criteria were (1) possession of a valid professional license from the Ministry of Health, (2) at least 1 year of clinical experience, and (3) direct involvement in patient care. Professionals in administrative roles or not directly engaged in patient management were excluded. A total of 300 invitations were distributed electronically, of which 270 completed responses were received (response rate 90%). The response rate was calculated at the overall survey level based on completed questionnaires relative to the total number of invitations distributed. The survey was distributed through professional online groups and networks of eye care clinicians. Because the questionnaire was not sent to a fixed list of individuals, accurate denominators for each professional group were not available; therefore, group-specific response rates could not be calculated. An a priori sample size estimation was conducted using G*Power (version 3.1) [22] for multiple linear regression analysis. Assuming a medium effect size (f^2^=0.15), an α level of .05, statistical power of 0.80, and up to 10 predictors, the minimum required sample size was 118 participants. The final sample of 270 respondents exceeded this requirement, providing adequate statistical power for the planned analyses.

A self-administered electronic questionnaire was developed following a review of existing validated surveys on assistive technology adoption [10-12,14,18,23]. The instrument comprised five domains: (1) awareness, (2) training exposure, (3) institutional support, (4) perceived barriers, and (5) adoption of electronic low vision aids (Table 1). Items were rated on a 5-point Likert scale (1=strongly disagree to 5=strongly agree). Optional open-ended questions were included at the end of the survey to allow participants to provide additional comments or perspectives not captured by the closed-ended items. Selected anonymized comments are presented in the Results section to illustrate key quantitative findings; no formal qualitative coding or thematic analysis was performed.

**Table 1.** Survey domains, representative items, and mean Likert scores (5-point scale; 1=strongly disagree, 5=strongly agree; N=270 respondents).

Domain and representative item		Score, mean (SD)
Awareness		
	I am familiar with the main types of electronic LVAs.^a^	3.6 (0.7)
	I understand the difference between optical and electronic devices.	3.5 (0.8)
	I know about digital magnifiers and smart glasses.	3.8 (0.8)
	I am aware of the benefits of electronic LVAs in improving reading and mobility.	3.7 (0.7)
	I have read or heard about electronic LVAs through professional sources.	3.4 (0.9)
	I know where to obtain information about new low vision technologies.	3.5 (0.8)
Training exposure		
	I attended workshops or training sessions on electronic LVAs.	2.7 (0.9)
	I received hands-on demonstration during my education.	2.8 (0.8)
	I participated in online webinars related to electronic LVAs.	2.9 (0.9)
	I practiced using electronic magnifiers with patients.	2.6 (0.9)
	I have self-trained using online tutorials or videos.	2.7 (0.8)
Institutional support		
	My workplace provides access to low vision devices for demonstration.	2.9 (0.8)
	My department encourages use of electronic aids in patient management.	3.0 (0.9)
	Institutional funding is available for electronic LVA training.	2.7 (0.9)
	My clinic has a designated low vision service or room.	2.8 (0.8)
	Technical support is available for device maintenance.	2.8 (0.9)
Perceived barriers		
	The high cost of devices limits their use in clinical practice.	4.2 (0.7)
	Lack of professional training prevents effective use of electronic LVAs.	3.9 (0.8)
	Limited institutional support restricts adoption.	4.1 (0.8)
	Low patient awareness	3.8 (0.8)
	There is a lack of Arabic-language software support.	3.7 (0.9)
	Devices are difficult to use for older adults or individuals with limited formal education.	3.9 (0.7)
	There is limited technical support for device maintenance.	3.6 (0.8)
	Import regulations make devices unavailable.	3.5 (0.9)
Adoption behavior		
	I currently prescribe or recommend electronic LVAs to my patients.	3.5 (0.8)
	I routinely demonstrate electronic LVAs in my clinic.	3.4 (0.8)
	I feel confident teaching patients how to use electronic LVAs.	3.6 (0.7)
	I intend to integrate electronic LVAs more frequently in future practice.	3.8 (0.8)
	I believe electronic LVAs improve my patients’ quality of life.	4.1 (0.7)

a LVA: low vision aid.

The survey was administered in English, as English is the primary language of instruction and clinical documentation among eye care professionals in Jordan. Participants were able to request clarification for any items if needed. The questionnaire underwent expert review by 3 senior vision rehabilitation specialists to establish content validity, followed by pilot testing with 25 clinicians. Pilot data were excluded from the final analysis. Minor linguistic refinements were made to improve clarity and internal consistency. Internal consistency was assessed using Cronbach α for each domain (Table 2). Values ≥0.70 were considered acceptable.

**Table 2.** Internal consistency (Cronbach α) of questionnaire domains based on the final survey sample (N=270).

Domain	Cronbach α
Awareness	0.86
Training exposure	0.81
Institutional support	0.83
Perceived barriers	0.88
Adoption behavior	0.79

Data were analyzed using SPSS software (version 29; IBM Corp). Descriptive statistics summarized participant characteristics and mean domain scores. Inferential analyses included chi-square tests to compare aid adoption rates across professional groups, independent-sample *t*-tests to compare training hours between adopters and nonadopters, and multivariable binary logistic regression to identify predictors of electronic low vision aid adoption. Multicollinearity was assessed using variance inflation factors (<2). Model fit was evaluated using the Hosmer-Lemeshow test (*P*>.05). Statistical significance was set at *P*<.05 (2-tailed).

Professional experience was assessed using a single self-reported item asking participants to indicate their years of clinical practice in eye care. Awareness of electronic and digital low vision aids was measured using a composite awareness score derived from multiple Likert scale items assessing familiarity with device types, indications, and clinical applications. Training exposure was assessed by asking participants whether they had received formal or informal training related to electronic LVAs (yes or no), as well as the total number of training hours completed.

Internal consistency was calculated using Cronbach α for each domain (Table 2). Values ≥0.70 were considered acceptable. The α coefficients were calculated using responses from the final survey participants. Expert review (n=3) was used solely for content validity and was not included in the final internal consistency analysis.

### Ethical Considerations

This study was approved by the Institutional Review Board of Jordan University of Science and Technology (IRB/642) and was conducted in accordance with the principles of the Declaration of Helsinki. Participation was voluntary, and electronic informed consent was obtained from all participants prior to survey initiation. The survey was administered anonymously through a secure online platform. No personally identifiable information (such as names, identification numbers, or contact details) was collected. Data were stored on password-protected devices accessible only to the principal investigator and were analyzed in aggregate form to ensure participant confidentiality. Measures were implemented to minimize duplicate submissions.

## Results

A total of 270 eye care professionals completed the survey, representing a 90% response rate. Participants included 156 optometrists (57.8%), 78 ophthalmologists (28.9%), and 36 low vision specialists (13.3%) working across multiple practice settings in Jordan. The mean age was 36 (SD 8) years, and 149 of 270 (55.2%) participants were female. Respondents reported a mean of 12 (SD 6) years of professional experience, indicating a clinically experienced cohort.

Across all participants, the mean awareness score regarding electronic LVAs was 3.2 (SD 0.7) on a 5-point Likert scale, suggesting moderate familiarity. The mean training exposure time was 18.4 (SD 12.5) hours. However, substantial variability was observed, with many clinicians reporting fewer than 10 hours of formal training. These data are summarized in Table 3.

**Table 3.** Participant characteristics (N=270).

Characteristic	Value
Profession, n (%)	
Optometrist	156 (57.8)
Ophthalmologist	78 (28.9)
Low vision specialist	36 (13.3)
Gender, n (%)	
Female	149 (55.2)
Male	121 (44.8)
Age (y), mean (SD)	36 (8)
Professional experience (y), mean (SD)	12 (6)
Awareness score (1-5), mean (SD)	3.2 (0.7)
Training hours, mean (SD)	18.4 (12.5)

Profession-specific analyses revealed that low vision specialists had the highest training exposure (mean 28.5, SD 9.2 hours) and awareness score (mean 3.8, SD 0.6), followed by optometrists (mean 17.6, SD 11.3 training hours; mean 3.3, SD 0.7 awareness score) and ophthalmologists (mean 12.1, SD 8.5 training hours; mean 3.0, SD 0.6 awareness score). Gender and years of experience were not significantly associated with awareness or training exposure (*P*>.05).

Among all respondents, 117 of 270 professionals (42.2%) reported that they currently prescribe or recommend electronic LVAs to patients. Adoption was significantly higher among low vision specialists (22/36, 61%) than optometrists (69/156, 44.2%) and ophthalmologists (26/78, 33%) (*χ*^2^=9.64*; P*=.008). Eye care professionals who reported active aid adoption also demonstrated substantially greater training exposure (mean 23.6, SD 11.2 hours) than nonadopters (mean 14.9, SD 9.8 hours), a difference that was statistically significant (*t*=5.74; *P*<.001). Additionally, adopters also had higher awareness scores than nonadopters (mean 3.6, SD 0.6 vs mean 3.0, SD 0.7; *P*<.001) and higher institutional support scores (mean 3.4, SD 0.8 vs mean 2.9, SD 0.7; *P*=.002).

Overall, these results suggest that professional experience alone does not drive aid adoption; rather, the presence of structured training and institutional support are key determinants of electronic LVA use. The perceived barriers domain comprised 8 Likert scale items representing 8 distinct barriers to electronic LVA adoption (Table 1). Respondents most frequently cited high device cost (213/270, 79%), followed by lack of training opportunities (184/270, 68.1%), limited institutional support (173/270, 64%), and low patient awareness (154/270, 57%; Table 4) as barriers to electronic LVA adoption.

**Table 4.** Barriers to electronic low vision aid adoption among Jordanian eye care professionals (N=270).

Perceived barriers	Respondents, n (%)
Import regulations make devices unavailable	111 (41.1)
There is limited technical support for device maintenance	124 (45.9)
Devices are difficult to use for older adults or individuals with limited formal education	130 (48.1)
There is a lack of Arabic-language software support	140 (51.9)
Low patient awareness	154 (57)
Limited institutional support restricts adoption	173 (64)
Lack of professional training prevents effective use of electronic low vision aids	184 (68.1)
The high cost of devices limits their use in clinical practice	213 (79)

Subgroup analysis revealed that cost concerns were particularly pronounced among optometrists (131/156, 84.0%), whereas low vision specialists reported greater concern regarding limited patient awareness (22/36, 61%) and lack of Arabic-language device interfaces (20/36, 56%). In contrast, ophthalmologists more frequently emphasized institutional limitations (56/78, 72%) and unclear referral mechanisms (50/78, 64%) as major obstacles.

One-way ANOVA demonstrated significant between-group differences for lack of professional training (*F*_2,267_=6.21; *P*=.002) and limited maintenance or technical support (*F*_2,267_=4.89*; P*=.008). Post hoc analyses indicated that optometrists perceived these barriers more strongly than ophthalmologists and low vision specialists. Although cost- and patient-related barriers were commonly reported across all professional groups, no statistically significant between-group differences were observed for these domains (Multimedia Appendix 1). Comments from participants reinforced these findings, as illustrated by the following representative quotes:

*Even when devices are available, few clinicians are trained to demonstrate them effectively.*

[Participant 47, optometrist]

*Cost remains prohibitive for many families, especially without Ministry of Health support.*

[Participant 112, ophthalmologist]

These results point to a multifactorial set of barriers (financial, educational, and systemic) that collectively hinder widespread electronic LVA integration into clinical practice.

A binary logistic regression model was fitted to identify predictors of electronic LVA adoption (Multimedia Appendix 2). The model explained 42% of the variance in adoption behavior (Nagelkerke *R*^2^=0.42) and correctly classified 79% of cases. Training hours emerged as the strongest positive predictor of electronic low vision aid adoption (odds ratio [OR] 1.82, 95% CI 1.31‐2.53; *P*<.001). Higher institutional support (OR 1.48, 95% CI 1.12‐1.96; *P*=.008) and greater awareness (OR 1.35, 95% CI 1.05‐1.72; *P*=.02) were also significantly associated with increased adoption. In contrast, high device cost was a strong negative predictor (OR 0.41, 95% CI 0.27‐0.62; *P*<.001), while lack of training was associated with a reduced likelihood of adoption (OR 0.65, 95% CI 0.48‐0.88; *P*=.006). Limited access was not a statistically significant predictor (OR 0.78, 95% CI 0.55‐1.08; *P*=.14).

**Figure 1.**
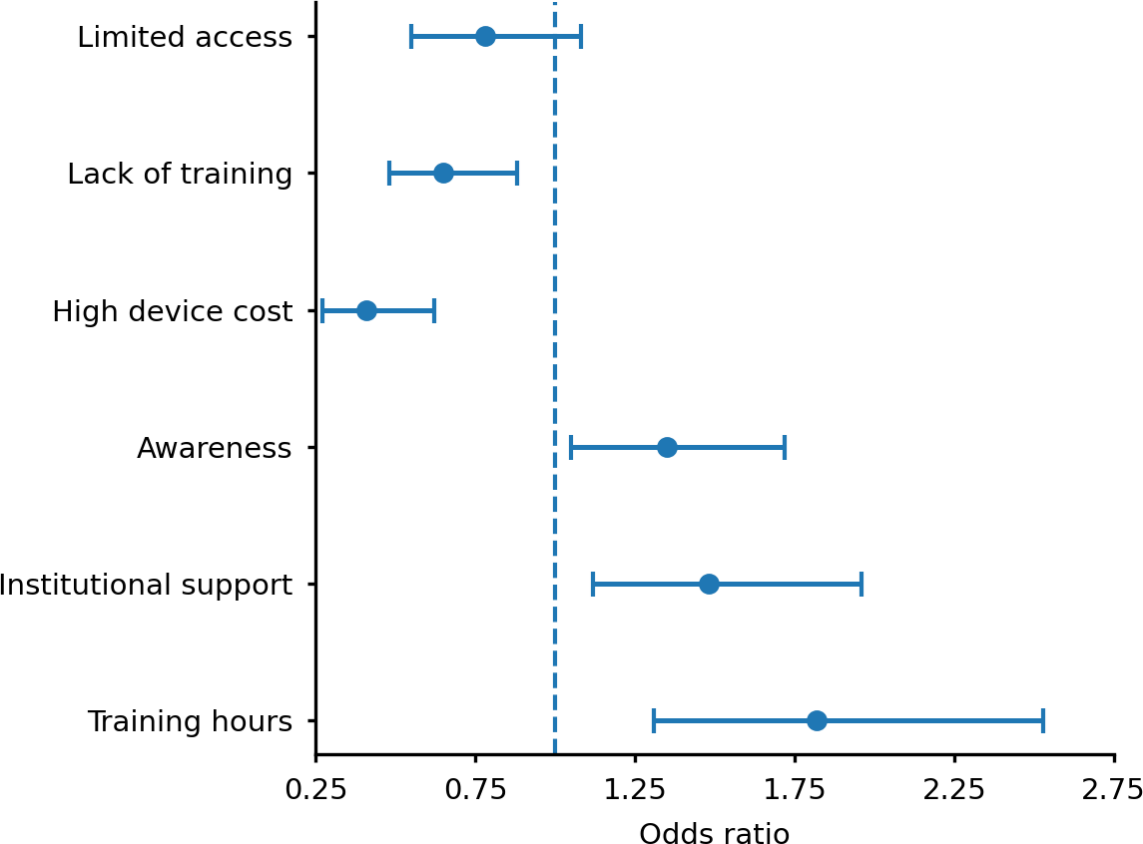
Forest plot showing adjusted odds ratios (with 95% CIs) for predictors of electronic low vision aid adoption.

Figure 1 illustrates the direction and strength of associations, showing that training exposure and institutional support exerted the most substantial positive influence, while high device cost exerted the strongest negative effect.

## Discussion

This study examined barriers to the adoption and recommendation of electronic LVAs among eye care professionals in Jordan. The main findings indicate that high device cost and lack of professional training were significantly associated with a lower likelihood of recommending electronic LVAs, whereas higher awareness and stronger institutional support were positively associated with adoption. Although overall awareness scores were moderate, fewer than half of respondents reported current use or recommendation of these devices, indicating a persistent gap between awareness and routine clinical implementation. These findings are consistent with reports from other low- and middle-income countries, where cost and training constraints are repeatedly identified as dominant barriers to electronic assistive technology uptake [10-12,14,18,23].

Multivariable regression analysis further demonstrated that training exposure, institutional support, and awareness independently predicted adoption of electronic LVAs, whereas perceived device cost was negatively associated with adoption. Together, these findings suggest that adoption is influenced by both individual-level factors (eg, professional knowledge and perceived benefit) and system-level factors (eg, organizational support and resource availability). This dual influence is consistent with prior research indicating that isolated improvements in clinician awareness are insufficient to promote sustained technology adoption without parallel investment in training and institutional infrastructure.

International studies report similar patterns of underuse. For example, surveys of eye care professionals in high-income settings have shown that fewer than half routinely recommend electronic or digital magnification devices despite recognizing their potential clinical benefit [13]. Studies from the Asia Pacific region and the Middle East likewise highlight limited institutional resources, high device cost, and insufficient professional training as persistent barriers to adoption [2,20]. These parallels suggest that the challenges identified in this study reflect broader implementation issues rather than context-specific resistance to technology.

Interpretation of these findings should also consider broader health system context in Jordan. Reports and policy analyses indicate that low vision rehabilitation services are often fragmented, with limited availability of designated low vision clinics, variable referral pathways, and uneven access to continuing professional education in rehabilitation technologies [1,12,24,25]. Although these system-level characteristics were not directly measured in this survey, they provide important context for understanding why institutional support and training emerged as prominent determinants of adoption. Global guidance from the World Health Organization emphasizes the need for integrated national rehabilitation frameworks to support equitable access to assistive and digital technologies, particularly in resource-constrained settings [3,20]. In this study, clinicians who perceived stronger institutional facilitation and greater patient benefit were more likely to recommend electronic LVAs, consistent with core constructs of the technology acceptance model and the unified theory of acceptance and use of technology framework [21].

The findings underscore the need for coordinated strategies to support electronic LVA adoption. Structured professional training in digital rehabilitation should be incorporated into undergraduate curricula and continuing professional development programs. Health care institutions may benefit from establishing demonstration or referral pathways that allow clinicians to trial devices with patients. In addition, funding mechanisms—such as subsidy schemes or public-private partnerships—may help mitigate cost barriers, particularly for older adults and children with visual impairment. These implications are aligned with international calls to strengthen rehabilitation systems and data collection under the World Health Organization Rehabilitation 2030 initiative [3].

Several limitations warrant consideration. The cross-sectional design precludes causal inference, and adoption behavior was assessed using self-reported measures rather than objective indicators such as prescription records or device use data. In addition, although the survey instrument was informed by established technology adoption frameworks, formal construct validity testing was not undertaken. This study also did not explicitly examine potential regional or socioeconomic variation within the eye care workforce, which may influence access to training, institutional support, and technology availability. These factors may limit generalizability and should be addressed in future research.

Future studies should use longitudinal or mixed methods designs to examine how training interventions, institutional policies, and funding mechanisms influence adoption over time. Incorporating validated measurement instruments, objective adoption indicators, and stratified analyses by region or practice setting would further strengthen the evidence base and inform targeted implementation strategies.

In conclusion, adoption of digital, smart, and electronic LVAs among eye care professionals in Jordan remains limited. Cost, training exposure, and institutional support were the primary factors associated with uptake in this study. These findings suggest that strengthening professional training opportunities, improving institutional facilitation, and exploring sustainable funding mechanisms may support broader integration of digital assistive technologies within low vision rehabilitation services. Greater coordination among clinicians, policymakers, and industry stakeholders may help address persistent implementation barriers and improve access to low vision care.

## Supplementary material

10.2196/87685 Multimedia Appendix 1 Subgroup analysis of mean barrier scores by professional category (N=270).

10.2196/87685 Multimedia Appendix 2 Logistic regression analysis for predictors of electronic LVA adoption.

10.2196/87685 Checklist 1 CHERRIES checklist.

## Abbreviations

CHERRIES: Checklist for the Reporting of Results of Internet E-Surveys
LVA: low vision aid
OR: odds ratio
