# Supplementary material for: Barriers to Adoption of Electronic Low Vision Aids Among Eye Care Professionals in Jordan: Descriptive Cross-Sectional Study
Source: JMIR Rehabil Assist Technol. 2026 Mar 2;13:e87685. doi: 10.2196/87685 (PMC12954482; doi:10.2196/87685)
Supplement: Multimedia Appendix 1 [file rehab-v13-e87685-s001.docx]

**Multimedia Appendix 1:** Subgroup analysis of mean barrier scores by professional category (N=270)

| **Barrier** | **Ophthalmologists (n=78) Mean (SD)** | **Optometrists (n=156) Mean (SD)** | **Low vision specialists (n=36) Mean (SD)** | **F (df=2, 267)** | ***P*-value** |
| --- | --- | --- | --- | --- | --- |
| High device cost | 4.43 (0.74) | 4.51 (0.62) | 4.46 (0.69) | 1.84 | .16 |
| Lack of professional training | 3.62 (0.85) | 4.12 (0.71) | 4.05 (0.68) | 6.21 | .002^a^ |
| Limited institutional support | 3.75 (0.81) | 4.02 (0.66) | 3.97 (0.64) | 3.17 | .046 ^a^ |
| Low patient awareness | 3.82 (0.77) | 3.97 (0.73) | 3.91 (0.75) | 2.09 | .13 |
| Lack of Arabic-language interface | 3.51 (0.88) | 3.63 (0.81) | 3.72 (0.79) | 1.24 | .29 |
| Device complexity (older users) | 3.43 (0.86) | 3.55 (0.74) | 3.61 (0.70) | 0.97 | .38 |
| Limited maintenance / technical support | 3.34 (0.82) | 3.78 (0.69) | 3.65 (0.73) | 4.89 | .008 ^a^ |
| Supply and import restrictions | 3.18 (0.93) | 3.24 (0.88) | 3.37 (0.81) | 1.12 | .33 |

^a^ Significant if *P*<.05
