# Supplementary material for: Barriers to Adoption of Electronic Low Vision Aids Among Eye Care Professionals in Jordan: Descriptive Cross-Sectional Study
Source: JMIR Rehabil Assist Technol. 2026 Mar 2;13:e87685. doi: 10.2196/87685 (PMC12954482; doi:10.2196/87685)
Supplement: Multimedia Appendix 2 [file rehab-v13-e87685-s002.docx]

**Multimedia Appendix 2:** Logistic regression analysis for predictors of electronic LVA adoption

| **Predictor** | **β (Coefficient)** | **SE** | **Wald χ²** | ***P*-value** | **Odds ratio (95% CI)** |
| --- | --- | --- | --- | --- | --- |
| Constant | –0.42 | 0.28 | 2.26 | .13 | — |
| Limited access | –0.25 | 0.17 | 2.17 | .14 | 0.78 (0.55–1.08) |
| Lack of training | –0.43 | 0.16 | 7.45 | .006^a^ | 0.65 (0.48–0.88) |
| High device cost | –0.89 | 0.21 | 17.8 | <.001^a^ | 0.41 (0.27–0.62) |
| Awareness | +0.30 | 0.12 | 5.9 | .02^a^ | 1.35 (1.05–1.72) |
| Institutional support | +0.39 | 0.14 | 7.1 | .008^a^ | 1.48 (1.12–1.96) |
| Training hours | +0.60 | 0.17 | 12.6 | <.001^a^ | 1.82 (1.31–2.53) |

^a^ Significant if *P*<.05. Model fit: (Nagelkerke R² =0.42; Hosmer–Lemeshow χ² = 7.83, *P*=.45; AUC=0.82 95% CI 0.77–0.87).
