# Supplementary material for: Barriers to Adoption of Electronic Low Vision Aids Among Eye Care Professionals in Jordan: Descriptive Cross-Sectional Study
Source: JMIR Rehabil Assist Technol. 2026 Mar 2;13:e87685. doi: 10.2196/87685 (PMC12954482; doi:10.2196/87685)
Supplement: Checklist 1 [file rehab-v13-e87685-s003.docx]

CHERRIES Checklist

| **Checklist category** | **Description** | **Location in manuscript** |
| --- | --- | --- |
| **Manuscript title** | Barriers to Adoption of Electronic Low Vision Aids Among Eye-Care Professionals in Jordan | Title page and the Manuscript |
| **Study type** | Descriptive cross-sectional online survey | Methods |
| **Survey design** | The study was conducted as an online cross-sectional survey targeting ophthalmologists, optometrists, and low vision specialists practicing in Jordan. | Methods |
| **IRB approval** | Ethical approval was obtained from the Institutional Review Board of Jordan University of Science and Technology. | Methods |
| **Informed consent** | Electronic informed consent was obtained from all participants prior to survey initiation. Participants were required to read an online consent statement and confirm agreement before accessing the questionnaire. | Methods |
| **Data protection** | Survey data were collected using a secure online platform and stored in password-protected files accessible only to the research team. | Methods |
| **Development and testing** | The questionnaire was developed based on a review of relevant literature and expert input in low vision rehabilitation. The survey was pilot-tested prior to dissemination to ensure clarity and relevance. | Methods |
| **Recruitment process** | This was a closed survey. Invitations were distributed electronically through established professional networks, including national professional associations, institutional mailing lists, and closed professional communication groups commonly used by eye-care professionals in Jordan. |  |
| **Survey administration** | The survey was administered online using a secure web-based platform. The questionnaire was available for a predefined data collection period. | Methods |
| **Response rate** | The response rate was calculated based on the number of completed questionnaires relative to the number of invitations distributed. The response rate was calculated at the overall survey level based on completed questionnaires relative to the total number of invitations distributed. Response rates were not calculated separately for each professional group. | Results |
| **Prevention of multiple entries** | To prevent duplicate submissions, responses were restricted to one submission per device or IP address. | Methods |
| **Handling of incomplete questionnaires** | Only fully completed questionnaires were included in the final analysis. Incomplete responses were excluded. | Methods |
| **Questionnaire order** | Survey items were presented in a fixed order. No randomization of items was applied. | Methods |
| **Adaptive questioning** | Adaptive questioning was not used. | Methods |
| **Statistical analysis** | Data were analyzed using appropriate statistical methods, including one-way ANOVA and multivariable logistic regression. | Methods, Results |
| **Incentives** | No incentives were offered for participation. | Methods |
| **Completeness checks** | The survey platform required completion of all mandatory closed-ended items before submission. Open-ended questions were optional, and responses to these items were not required for survey completion. | Methods |
